# Assessment the using of silica nanoparticles (SiO_2_NPs) biosynthesized from rice husks by *Trichoderma harzianum* MF780864 as water lead adsorbent for immune status of Nile tilapia (*Oreochromis niloticus)*

**DOI:** 10.1016/j.sjbs.2021.05.027

**Published:** 2021-05-21

**Authors:** Nashwa El-Gazzar, Taghreed N. Almanaa, Rasha M. Reda, M.N. El Gaafary, A.A. Rashwan, Fatma Mahsoub

**Affiliations:** a Department of Botany and Microbiology, Faculty of science, Zagazig University, 44519 Zagazig, Egypt; b Department of Botany and Microbiology, College of Science, King Saud University, 11495 Riyadh, Saudi Arabia; c Faculty of Veterinary Medicine, Department of Fish Diseases and Management, Zagazig University, 44511 Zagazig, Egypt; d Faculty of Technology and Development, Department of Animal & Poultry Production, Zagazig University, 44519 Zagazig, Egypt

**Keywords:** *Trichoderma harzianum*, Silica nanoparticles, Heavy metal, Nano-adsorbent, *Oreochromis niloticus*, IL-1*β* immune related gene expression

## Abstract

Rice husks (RHs) was used as a substrate for biosynthesis of high-value Silica nanoparticles (SiO_2_NPs). An isolate of *Trichoderma harzianum* MF780864 (*T. harzianum*) was isolated and identified based on the Internal Transcribed Spacers (ITS) sequences; it showed the potentiality to induce SiO_2_NPs in the process of RHs biotransformation. SiO_2_NPs were produced extracellularly and their size was of about 89 nm. SiO_2_NPs characterized by oval, rod and cubical particles by using Transmission Electron Microscope (TEM).The Fourier transform infrared spectroscopy (FTIR) confirmed the presence of various functional groups of biomolecules and capping protein, encapsulating SiO_2_NPs. Water and fish samples were collected from private fish farms in El-Sharkia Governorate, Egypt. Lead (Pb) was detected from water and fish samples at its highest concentration at about 0.088 mg/L. The adsorption capacity of Pb by SiO_2_NPs was evaluated by testing different concentrations of SiO_2_NPs viz. 1, 2, and 3 mg/L, wherein 1 mg/L revealed the highest Pb adsorption efficiency. Within laboratory trials, the results indicated that highest Pb adsorption efficiency revealed through the increasing of SiO_2_NPs concentrations until 120 h. *In vivo* trial that lasted for 8 weeks, Nile tilapia (*Oreochromis niloticus)* (29.78 ± 0.36 g body weight) supplemented with 0.088 mg/L Pb was divided into four experimental groups having three replicates (15 fish/replicate; 45 fish/group). The results showed that SiO_2_NPs supplementation through water revealed significant increase in growth and hematological parameters of *O. niloticus*. Moreover, enhancement of antioxidant capacity (TAC), and immune related gene expression of IL-1*β* were increased in the presence of SiO_2_NPs compared with the groups of Pb exposure. Moreover, Pb residue level in fish muscles was noticeably decreased in the SiO_2_NPs treated groups. Thus, this research opens up other possibilities in the field of using SiO_2_NPs as a lead adsorbent for water bioremediation.

## 1 Introduction

Aquatic systems cover approximately 71% of the Earth’s surface (Selim and Reda, 2015). In Egypt, Bahr El-Baqar drain is one of the largest polluted drains by agricultural, industrial, and sewage water, which passes through four highest densely populated Egyptian Governorates, such as Dakahlia, Sharkia, Ismailia, and Port-Said, and their wastewater drain into the Lake Manzala, North Egypt (Hamed et al., 2011). This may render the water to be unsuitable for household use, irrigation, fish cultivation and may adversely affect the aquatic life forms as fish are commonly considered as bio-indicators for heavy metals contamination in aquatic ecosystems (Kotsyuda et al., 2017, Bosch et al., 2016).

Heavy metals (HMs) contaminations are a matter of risk because of their toxicity, extended constancy and bioaccumulation quality (Rahman et al., 2012). Lead is one of the heavy metals that are often found in industrial wastewater from various industrial activities (Yarkandi, 2014, Vasanthi et al., 2019). Its discharge into the environment possess a serious threat due to its toxicity to aquatic and terrestrial lives (Thabede et al., 2020). Cadmium (Cd), lead (Pb), and mercury (Hg) are the most dangerous HMs, because of their cumulative nature in several organs (Seiler and Berendonk, 2012).

In this regard, there is no recommended method that has been yet detected for complete HMs removal. Therefore HMs toxicity is a world health problem and there is a need to continue research to find out novel ways to find out non-traditional and novel protocols for overcoming HMs toxicity. Some low cost adsorbents are used which indicate that adsorbents were outstanding removal capabilities for lead in aqueous solution such as RHs removal of lead (Abdel-Ghani et al., 2007).

RHs are an agricultural waste material and its annual worldwide production is approximately 500 million metric tons, of which 10–20% is rice husk. Dry RHs contain70–85% of organic matter and the remainder consists of silica, which is present in the cellular membrane (Premalal et al., 2002). Recently great attention has been focused towards using the bio-transformed RHs by fungi into SiO_2_NPs as an adsorbent for the removal of HMs pollutants from waste water. Thus, this study is an endeavor to improve the bioremediation of lead toxicity and immune status of *O. niloticus* using SiO_2_NPs*.*

Recent perspectives all over the world are to use safe materials as either safe therapy or as a biosorptive tools for toxic environmental wastes (Enan, 2006, Enan et al., 2013a, Enan et al., 2013b, Abdel-Shafi et al., 2016, Abdel-Shafi et al., 2019a, Abdel-Shafi et al., 2019b, Abdel-Shafi et al., 2019c, Abdel-Shafi et al., 2019d, Osman et al., 2020, Al-Mohammadi et al., 2020, Osman et al., 2021). In this framework, nanotechnology offers the ability to control matter at the nanoscale to have unique physical or chemical merits due to their small diameter, morphology or large surface area (El-Gazzar and Enan, 2020, El-Gazzar and Ismail, 2020). Nanotechnology is also used to prevent the formation of pollutants to treat and remediate contaminated water or contaminants by applying the material technology, industrial processes and others (El-Gazzar et al., 2020). Biosyntheses applied by fungi might appear attractive for nanoparticles in wider rate because they have a potentiality with standing during different conditions of bioprocesses due to their filamentous nature (Kar et al., 2014). Moreover, they are excessively confirmed to apply their mechanisms in biosynthesis of nanoparticles (Abdel-Salam et al., 2001, El-Gazzar and Ismail, 2020). The mediation process by fungal mycelium exhibited an excellent biocatalyst that applied in the biosynthesis of SiO_2_NPs with various application possibilities in different fields of medicine, engineering and environmental technology (Zielonka and Klimek-Ochab, 2017).

SiO_2_NPs diameters and their morphological aspects are variable parameters based on their production methods (El-Gazzar and Enan, 2020). In nature, SiO_2_NPs is deposited in phytoliths, which are siliceous bodies output by plant cells and accumulated or mineralized silica which is located in plant residues. They are interesting research tools due to their stability in plant tissues even after the organic parts are completely degraded (Dabney et al., 2016). In this regard, the present study was designed to (i) biosynthesis of SiO_2_NPs from RHs by *T. harzianum* MF780864 (ii) evaluate the highest concentrations of HMs in water and fish samples (iii) investigate the adsorption effect of silica in rice husk at Nano scale for heavy metals removal (iiii) enhance the immune status of *O. niloticus.*

## 2 Materials and methods

### 2.1 Rice husks (RHs)

RHs were collected from a local area (Abbassa, Abo-Hammad, Sharqia, Egypt). They were washed with distilled water many times, dried in a dry-oven (GCA, model 18EM, Precision Scientific group, Chicago, Illinois, USA) at 85 °C.

### 2.2 Isolation and characterization of fungal isolates

About 18 fungal isolates were isolated onto potato dextrose agar from soils contaminated with wastes from Ceramics and Photographic industries at local Egyptian area at 10th of Ramadan, (20 Km north Cairo, Egypt) (El-Gazzar and Ismail, 2020, El-Gazzar and Rabie, 2018). These fungal isolates obtained were screened for their ability to biosynthesis SiO_2_NPs using RHs solution as a substrate (El-Gazzar and Ismail, 2020, El-Gazzar et al., 2020). Only one isolate no.13 showed the potentiality to do so. This fungal isolate no.13 was characterized morphologically at the genus level as described previously (El-Gazzar and Rabie, 2018, Ellis et al., 2007). For identification of the isolate no.13 at the species level, molecular characterization of 18S rRNA gene was used (Ellis et al., 2007). Briefly, the isolate no.13 was grown in sterile Petri plates containing autoclaved Sabouraud‘s Dextrose Agar (SDA) medium and incubated for 7 days at 28 °C (Ellis et al., 2007). Cultures were sent to the Molecular Biology Research Unit, Assiut University for DNA extraction using Patho-gene-spin DNA/RNA extraction kit provided by Intron Biotechnology Company, Korea. The fungal DNA was then sent to SolGent Company, Daejeon South Korea for polymerase chain reaction (PCR) and rRNA gene sequencing (El-sayed et al., 2015). PCR was performed using ITS1 (forward) and ITS4 (reverse) primers which were incorporated in the reaction mixture. Primers have the following composition: ITS1 (5′ - TCC GTA GGT GAA CCT GCG G − 3′), and ITS4 (5′- TCC TCC GCT TAT TGA TAT GC −3′). The purified PCR products (amplicons) were sequenced with the same primers (White et al., 1990). The obtained sequences were analyzed using Basic Local Alignment Search Tool (BLAST) from the National Center of Biotechnology Information (NCBI) website. Phylogenetic analysis of sequences was done with the help of MegAlign (DNA Star) software version 5.05.

### 2.3 Biosynthesis of SiO_2_NPs

Preliminary fungal biomass was prepared as a biocatalyst for biosynthesis of SiO_2_NPs. To obtain this biomass (biocatalyst) the fungal strain was cultured into Czapek- Dox liquid medium containing per liter: 30 g sucrose, 0.5 g mgSO_4_ * 7 H_2_O, 0.5 g KCl, 2.64 g (NH_4_)_2_SO_4_, 0.01 g FeSO_4_ and 0.5 g K_2_HPO_4_, pH 7.2(El-Gazzar and Rabie, 2018). Cultures were grown in a rotary shaker at (130 rpm) in 250 mL Erlenmeyer flasks containing 100 mL medium, which was inoculated with spores suspension in 0.05% Triton X-100 to a density of 10 000 spores mL^−1^ and incubated at 27 °C until mid-log phase (4 days). Then, the mycelium was separated by filtration, washed twice with distilled water and finally suspended in 100 mL sterile water. After 24 h of incubation under starvation conditions, the mycelium was separated by filtration and was used as a biocatalyst.

The biosynthesis of SiO_2_NPs was done by the following protocol described previously (El-Gazzar et al., 2020) with some modification. About 4 g of raw RHs were suspended in 100 mL of the biotransformation medium de-ionized water and the biocatalyst (10 g of wet fungal biomass) was added. Then, these biotransformation flasks were incubated on a rotary shaker at 130 rpm for a period of 16 days. At the same time, corresponding control experiments lacking the biocatalyst were carried out. Then the post-biotransformation fluid was separated from the substrate by gravity filtration on a qualitative disc filter with pore size 240 mm. Post-biotransformation fluid was transferred to Petri dishes and dried in laboratory drier (200 °C for 2 h). The concentration and physical properties of the developed SiO_2_NPs were determined as described below.

### 2.4 Characterization of the biosynthesized SiO_2_NPs

The physical properties of the prepared post-biotransformation SiO_2_NPs were determined by the following characterization methods:

#### 2.4.1 UV–visible spectrophotometer

The prepared post-biotransformation fluid was monitored by UV–visible spectrophotometer (T80 + UV Flash spectrophotometer PG Instrument LTD), (DeAlba-Montero et al., 2017).

#### 2.4.2 Dynamic light scattering system (DLS)

The DLS was generally used for analysis of the size distribution pattern of particles in suspension or solution which gives the hydrodynamic diameter of particles (Archna, 2016). DLS (Malvern, UK) was carried out at Regional Center for Food and Feed, Agriculture Research Centre (ARC), Giza, Egypt.

#### 2.4.3 Transmission electron microscope analysis (TEM)

The morphology and size of the biosynthesized SiO_2_NPs were characterized by TEM operated at 100KV connected with CD camera, Japan(Jain et al., 2011, Abdel-Shafi et al., 2020).

#### 2.4.4 Fourier transform infrared spectroscopy (FTIR)

The characterization of functional groups on the surface of nanoparticles was performed by FTIR (Thermo Nicolet model 6700 spectrum Located at Micro-Analytical Center, Cairo University, Giza, Egypt) by employing KBr pellet technique and the spectra were scanned in the 400–4000 cm^−1^ range at a resolution of 4 cm^−1^(Enan et al., 2020, El-Sayed et al., 2020).

### 2.5 Collection of water and fish samples for heavy metal content analysis

Water and fish samples were collected from the most important sites for fish aquaculture in the Sharkia Governorate in Egypt, Sahl Al Hussainia fish farms (2 Km south the Mediterranean sea) that receive their water supply from Bahr El-Baqar drain, Egypt. Three water and *O. niloticus* samples were randomly collected from each pond (nine water and fish samples/site). The tested HMs were Cd, As, Cu, Fe, Hg, Pb, Mn and Zn. Water samples were also tested to determine the Cd, As, Cu, Fe, Hg, Pb, Mn and Zn content using an atomic absorption spectrophotometer (model 210VGP,Buck Scientific USA). Also, the fish muscle HM contents were determined using an atomic absorption spectrophotometer (model 210VGP, Buck Scientific, USA).

### 2.6 The in vitro lead adsorbent by SiO_2_NPS

Nine aquaria were filled with water. According to the water samples analyzed from different fish farms; the highest dose of Pb found in this analyzed water samples at 0.088 mg/L. Lead (Pb): Lead acetate trihydrate salt (C_4_ H_6_ O_4_ Pb_3_ H_2_O) was obtained from Hi-lab for Trading Chemicals and Medical Appliances, Sharkia, Egypt. Lead at 0.088 mg/L was added to the aquarium water to examine the adsorption capacity of SiO_2_NPs.Thereafter, 1, 2 and 3 mg of SiO_2_NPs were weighed and were added to each liter of aquarium water (3 aquaria/each concentration).Water samples were taken after 0, 24, 48, 72, 120 and 168 h to determine the adsorption capacity of SiO_2_NPs to Pb, which was analyzed using an atomic absorption photometer.

### 2.7 Atomic absorption spectrophotometer (A.A.S)

Quantitative determination of lead concentration in water samples was carried out by “Buck scientific (Model 210VGP, Buck Scientific USA) (n = 3/treatment). Atomic Absorption Spectrophotometer” was carried out in the Faculty of Veterinary Medicine, Zagazig University, Egypt.

### 2.8 Experimental design for evaluation of SiO_2_NPs adsorption capacity in vivo

A sample of apparently healthy *O. niloticus* (average weight 29.78 ± 0.36 g) was tested and each 15 fish samples were acclimated in glass aquaria (80 × 40 × 30 cm) filled with 60 L of dechlorinated tap water for 15 days and fed on a basal diet before the initiation of the experiment according to the slandered requirements of *O.niloticus* at the Fish Research Unit of the Faculty of Veterinary Medicine, Zagazig University, according to the standard experimental fish requirements (NRC, 2011). The experiment lasted for 60 days during which, *O. niloticus* samples were randomly divided into four groups, each group having three replicates (15 fish/replicate; 45 fish/group). The 1st group served as a control group without any treatment in water. The 2nd group (SiO_2_NPs) supplemented with 1 mg/L SiO_2_NPs in water; the 3rd group was supplemented with 0.088 mg/L (Pb) in water; the fourth group (SiO_2_NPs + Pb) supplemented 0.088 mg/L (Pb) and 1 mg/L nano in water. Water was completely replaced every 48 h. *O. niloticus* samples were fed twice daily (9:00 a.m. and 3:00p.m.) at a rate of 3% of their biomass. Throughout the experimental period (60 d), the water quality parameters were monitored and maintained within the appropriate range in the aquaria (APHA, 1998) as following: temperature 28 ± 1.02 °C, pH 6.9 ± 0.1, dissolved oxygen 7.4 ± 0.34 mg/L, ammonia 0.035 ± 0.01 mg/L, nitrite 0.03 ± 0.010 mg/L, total hardness 141 ± 1.2 mg/L, total dissolved solids 230 ± 2 mg/L, conductivity 370 ± 2.7 μS/cm, Ca^+2^ 33 ± 0.12 mg/L, Mg^+2^ 19 ± 0.25 mg/L, Na^+^ 10 ± 0.13 mg/L, K^+^ 2.7 ± 0.002 mg/L, HCO^-^_3_ 96.50 ± 2.5 mg/L, SO_4_^2−^ 52 ± 1.3 mg/L and Cl^−^ 18 ± 0.7 mg/L. Water and Pb have been changed completely regularly and replaced with freshly prepared Pb solution. The actual Pb level was measured twice daily using atomic absorption photometer (model 210VGP, Buck Scientific USA) before and after water renewal and addition of freshly prepared Pb solution. The Pb levels before and after water renewal was 0.088 ± 0.00230.mg L^−1^ and 0.090 ± 0.00305 mg L^−1^, respectively (n = 9/Treatment). All procedures of the current experiment were carried out in accordance with the Egyptian laws and university guidelines for the care of experimental animals and have been approved by the Committee of the Faculty of Veterinary Medicine, Zagazig University, Egypt. *O. niloticus* behavior changes and mortality rate were recorded throughout the experimental period.

### 2.9 Analytical parameters of the experimental study for evaluation of SiO_2_NPs adsorption capacity

#### 2.9.1 Growth parameters

To determine the growth efficiency, the fish was weighed every two weeks, which was calculated according to the following equations (Selim and Reda, 2015):

$$\text{Weight}\;\text{gain}\;\left(\text{WG}\right)=\text{final}\;\text{body}\;\text{weight}\left(\text{FBW}\right)-\text{initial}\;\text{body}\;\text{weight}\left(\text{IBW}\right)\;$$

$$\text{Specific}\;\text{growth}\;\text{rate}\left(\text{SGR}\right)=\frac{FBW-IBW}{experimental\;day}x\;100$$

$$\text{Food}\;\text{conversion}\;\text{ratio}\left(\text{FCR}\right)=\frac{Total\;feed\;intake}{WG}$$

#### 2.9.2 Blood sample collection and hematological index analysis

Three blood samples/replicate (nine blood samples/group) were collected from caudal vein of fish by using a 1 mL heparinized syringe at the end of feeding period (60 d). For calculating hematocrit value (Hct; %), blood was centrifuged at 10,000xg for 5 min. The total erythrocytes (RBCs; 10^6^/mL) were counted by using hemocytometer (El-Gazzar and Ismail, 2020). The hemoglobin concentration (Hb; g/dl) was assayed by cyanmethemoglobin method (Groff and Zinkl, 1999). Mean corpuscular hemoglobin volume (MCV; fl), mean cell hemoglobin (MCH; pg), and mean corpuscular hemoglobin concentration (MCHC; g/dl) were calculated according to (Lewandrowski, 2015). Total number of white blood cells (WBCs; 10^3^/µl) and differential counts were estimated manually according to the method described previously (Stoskopf, 1993).

#### 2.9.3 Collection of serum samples and biochemical, immunologic, and antioxidant parameter analysis

Three blood samples/replicate (nine blood samples/group) were collected from caudal vein of O. niloticus and transferred into Eppendorf tubes without anticoagulant and centrifuged at 3000 rpm for 15 min. The colorimetric method was used for estimating liver enzyme activity for each of sample alanine aminotransferase (ALT, U/L), aspartate aminotransferase (AST, U/L) and alkaline phosphatase (ALP, U/L) according to the description of Reitman (Reitman and Frankel, 1957). Regarding the assessment of the kidney function, the levels of urea and creatinine were calculated by the methods described previously (Coulombe and Favreau, 1963, Larsen, 1972). The nitric oxide (NO) activity was measured by using Griess reagent method (Green et al., 1982), while turbidity assay by Micrococcus lysodekticus were used to determine the lysozyme activity (LYZ) (Ellis, 1990). The total activity of antiprotease (APA) was determined by the capacity of the serum to inhibit trypsin activity (Hanif et al., 2004). Oxidant and antioxidant stress were determined by analyze total antioxidant capacity (TAC, mM/L), Superoxide dismutase (SOD, U/mL) and Malondialdehyde (MDA, nmol/mL) as the manufacturer of commercial colorimetric kits (Biodiagnostic Co., Egypt) described.

#### 2.9.4 Collection of spleen samples and determination of immune related cytokines IL-1ß expression

At the end of feeding period (60 d), three spleen samples/replicate (9 samples/group) were collected according to the manufacturer's procedure. Complete RNA was extracted from the spleen tissue using aRNeasy Mini Kit (Qiagen, Heidelberg, Germany). The spectrophotometer was used to evaluate the quality and concentration of RNA. Elongation factor 1 alpha (EF-1 alpha) (F: 5′-GCTTCAACGCTCAGGTCATC-3′; R: 5′-TGTGGGCAGTGTGGCAATC-3′) served as a housekeeping gene (Gröner et al., 2015). The primers for the target immune gene (*IL-1β*) were (F: 5′-TGCTGAGCACAGAATTCCAG-3′; R: 5′-GCTGTGG AGAAGAACCAAGC-3′) (Pirarat et al., 2011). The thermal cycling conditions used were initial denaturation at 94 °C for 5 min, followed by 40 cycles of amplification (DNA denaturation at 94 °C for 15 s, annealing at 62 °C for 30 s, extension at 72 °C for 30 s), and final extension at 62 °C for 1 min. The amplification of the examined gene was evaluated by creating melting curve and the standard curve was performed to determine the amplification efficiency of the used primer. Amplification efficiencies were above 97%.The level of immune related *il-1β* was expressed by relative fold changes calculated by ^2−ΔΔ^CT in which the CT of each sample was compared with the control group (Livak and Schmittgen, 2001).

### 2.10 Determination of residual Pb level in fish muscles

Three *O. niloticus* samples/replicate (9 samples/group) were taken from Pb and Pb + SiO_2_NPs groups and analyzed at the end of the experiment using an atomic absorption photometer (model 210VGP, Buck Scientific, USA).

### Ethical Approval

All procedures involving animals performed in the study were performed in accordance with the ethical standards of our university by ZU-IACUC committee. Approval number ZU-IACUC/2/F/7/2021.

### 2.12 Statistical analyses

The data obtained were subjected to one-way ANOVA; Post hoc: Duncan’s multiple comparisons, to determine the significant variations among the various parameters in the experimental groups. All of the statistical analyses were performed using SPSS version 14 (SPSS, Chicago, IL, USA). A P value of < 0.05 was considered statistically significant.

$${\text{Y}}_{\text{ij}}=\mu +{\text{R}}_{\text{i}}{\text{+e}}_{\text{j}}$$

Where: Y_ij_ = An observation, µ=Overall mean, R_i_ = Remediation by SiO_2_NPs (i = 1), and e_j_ = Experimental errors.

## 3 Results

### 3.1 Potency of the fungal isolate for SiO_2_NPs biosynthesis

**Fig. 1:**
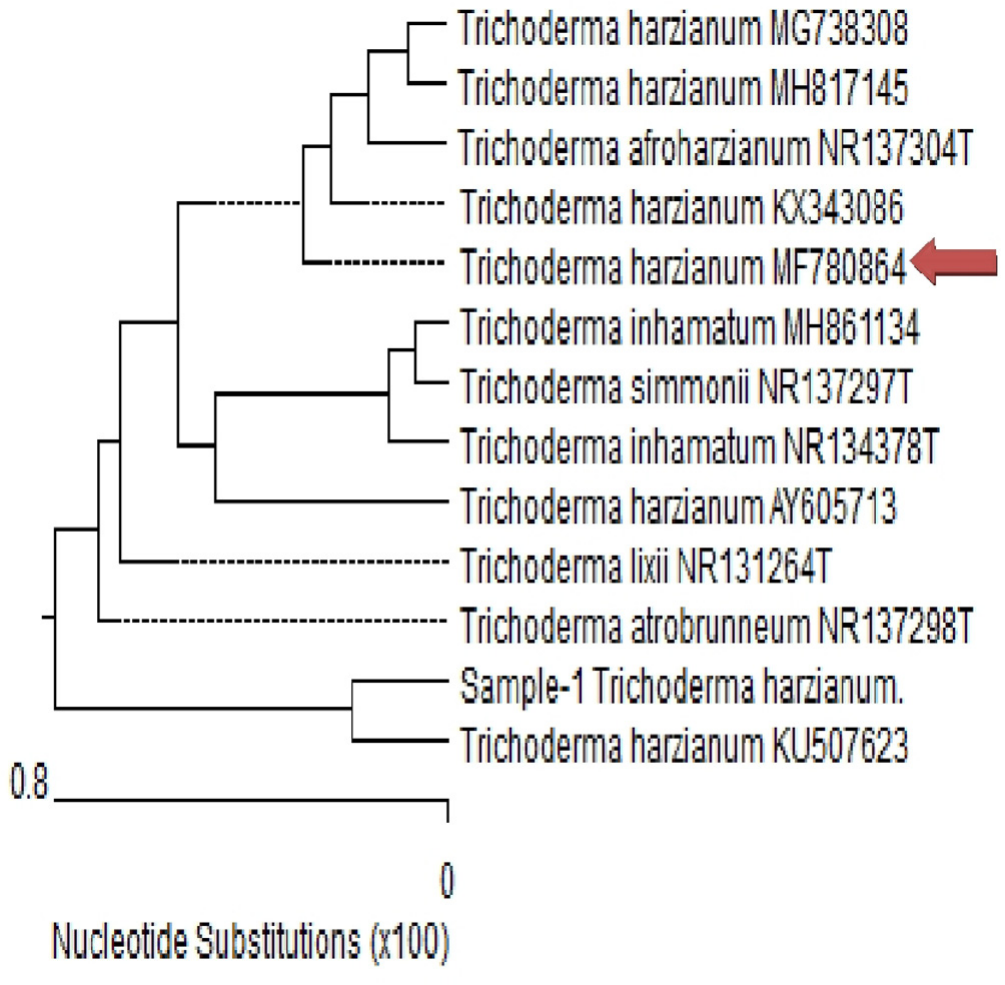
Phylogenetic tree based on ITS sequences of 18S-28S rRNA of the fungal strain (sample −1 Trichoderma harzianum. MF780864) isolated in the present study aligned with closely related sequences accessed from the Gen Bank.

Out of 18 fungal isolates obtained, only one no.13 appeared to biosynthesize SiO_2_NPs as appeared by the instrumental analysis (UV–Vis, TEM and DLS) carried out herein. This isolate no.13 was identified at genus level morphologically as *Trichoderma sp*. For species identification, molecular characterization was carried out for 18S-28S rRNA gene sequence. DNA was extracted from *Trichoderma* sp (no.13). The PCR test was carried out for the 18S-28S rRNA gene. The PCR product was electrophoresed in 1% agarose gel and showed DNA band of about 593 bp. The amplicon of PCR 18S-28S rRNA product flanking ITS1,2 and 5.8 S region for *Trichoderma sp* was sequenced (Supplementary Fig. 2). Molecular and morphological studies collectively approve the fungal isolates as *Trichoderm sp* with accession number MF780864. From the alignment profile, *Trichoderma s*p. (sample-1) showed 99.49% identity with *Trichoderma harzianum* KU507623 with percentage coverage of 99%. It showed also 99.82% identity with the type strain of *Trichoderma trobrunneum*NR137298T (Fig. 1). *Trichoderma harzianum* strain ACCC32857 18S ribosomal RNA gene, partial sequence; internal transcribed spacer 1, 5.8S ribosomal RNA gene, and internal transcribed spacer 2, complete sequence; and 28S ribosomal RNA gene, partial sequence. The isolate in the present study aligned with closely related sequences accessed from the Gen Bank. Hence, the isolate of *Trichoderma harzianum* was designed as FM- *Trichoderma harzianum* MF780864. (Fig. 1).

### 3.2 Characterization of SiO_2_NPs

**Fig. 2:**
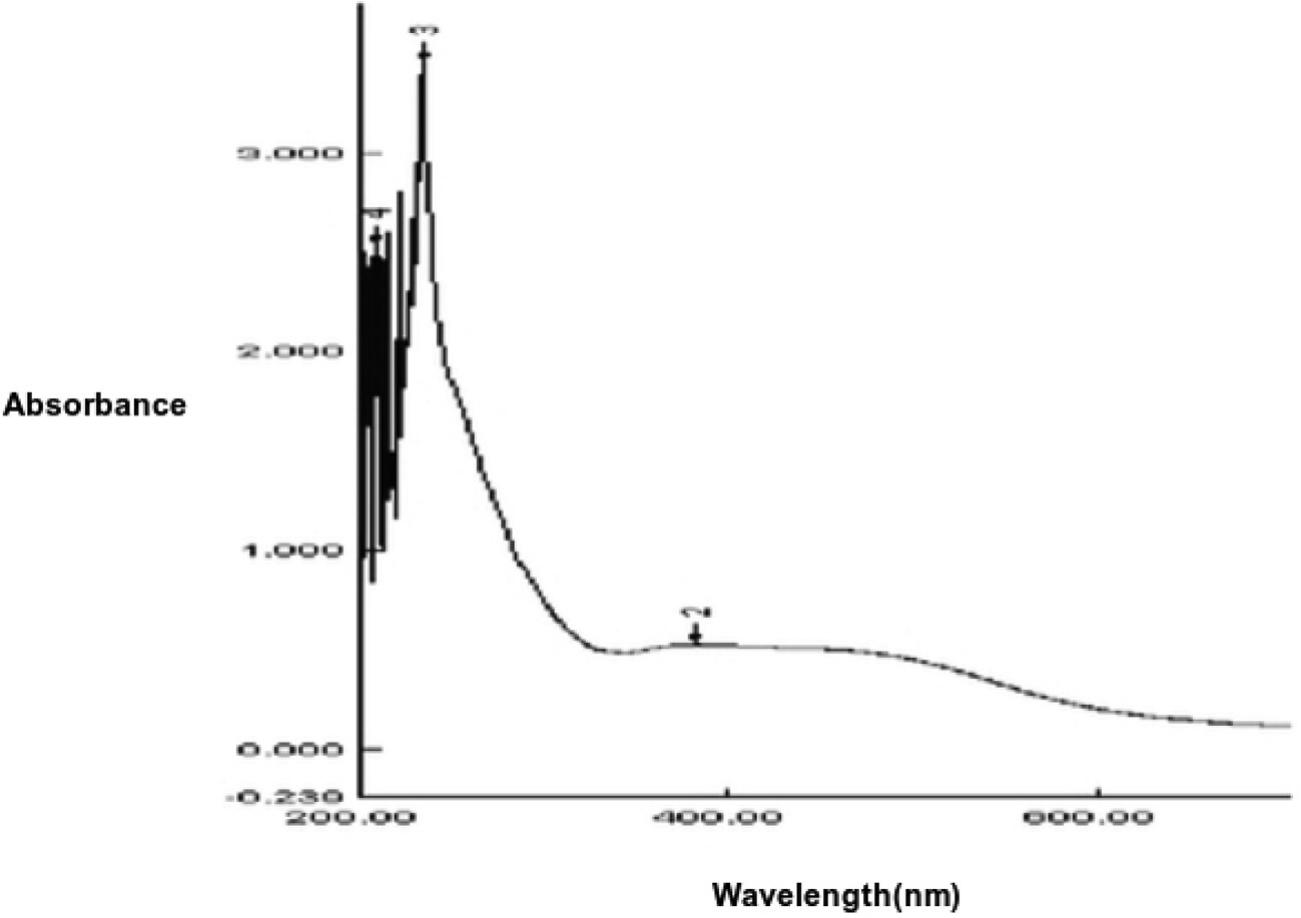
The UV–Visible spectrum showing the absorption peak of SiO_2_NPs biosynthesized by *T. Harzianum* at 336 nm.

**Fig. 3:**
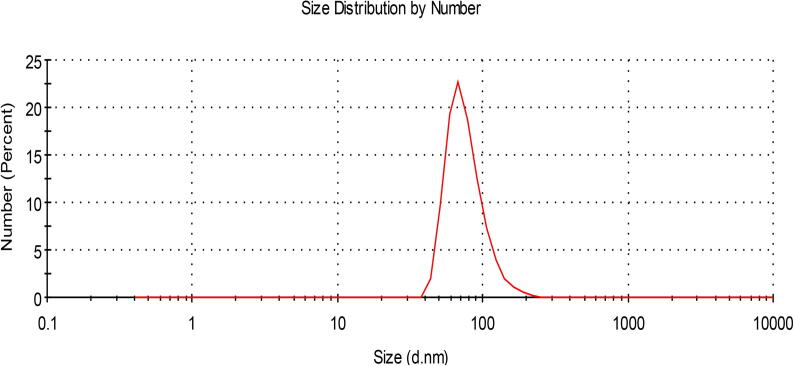
Histogram shows particle size distribution analyzed by Dynamic light scattering system (DLS); an average particle size based on intensity distribution for SiO_2_NPs at 89 nm.

**Fig. 4:**
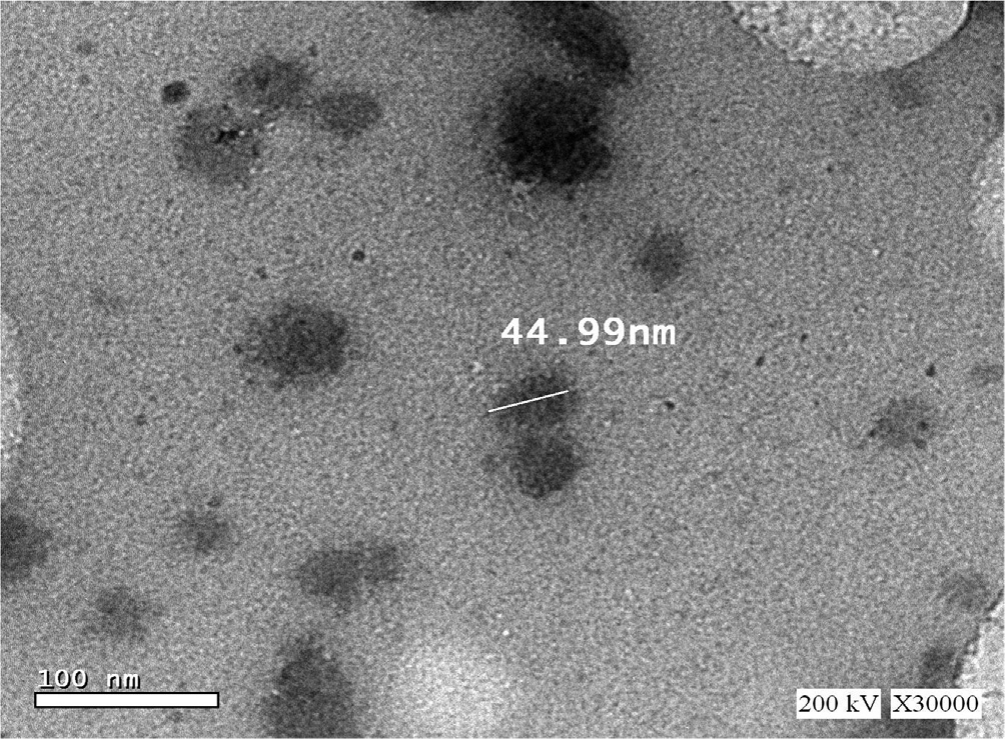
The shape and size of SiO_2_NPs under Transmission Electron Microscope (TEM) showing oval, cubic and rod shapes with 6.49 nm.

**Fig. 5:**
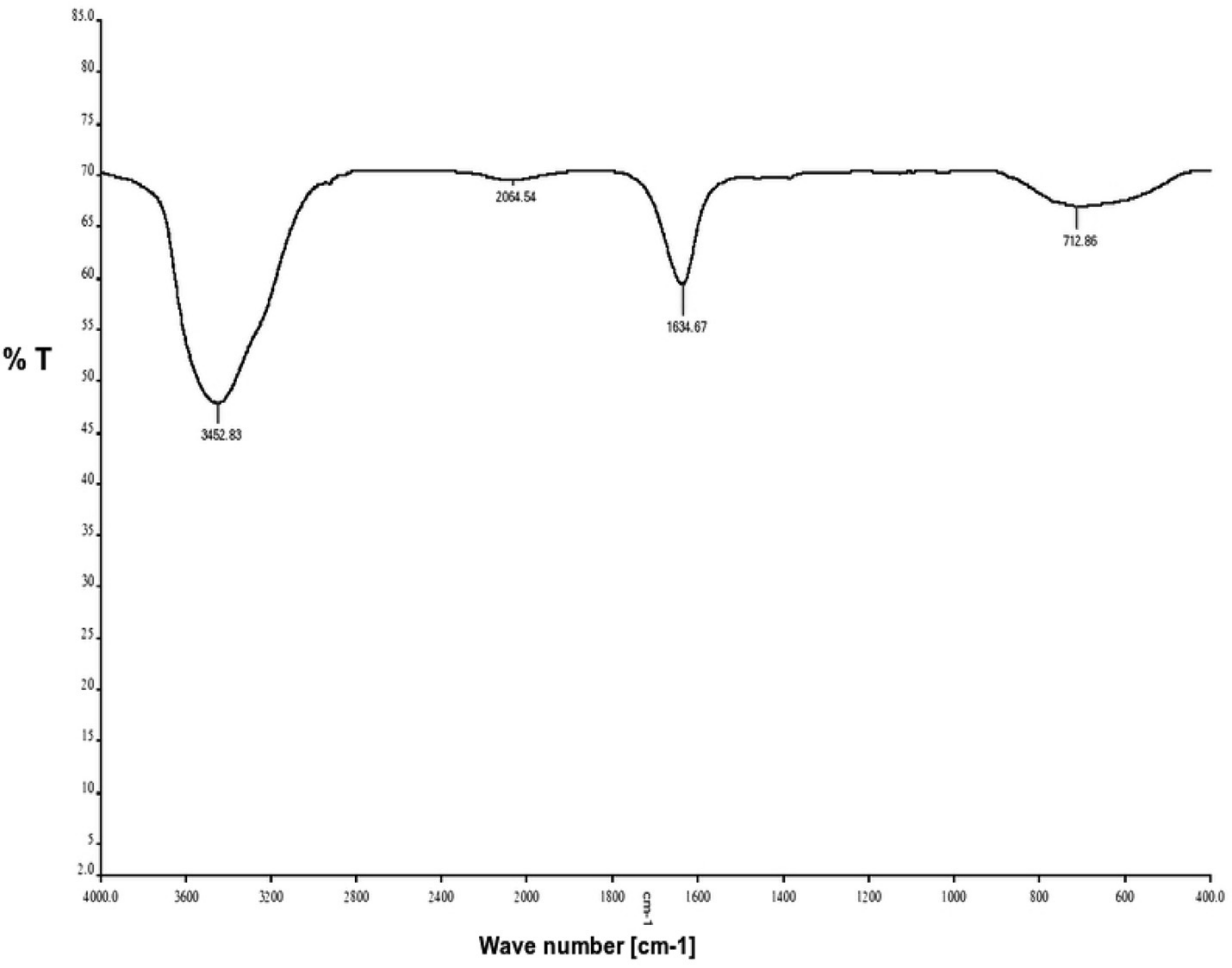
Characterization of functional groups on the surface of SiO_2_NPs by Fourier transform infrared spectroscopy (FTIR) spectrum.

SiO_2_NPs had maximum absorption at about 336 nm when subjected to UV–visible spectral scan corresponding to surface Plasmon resonance that indicating the formation of SiO_2_NPs (Fig. 2). Particle size distribution analyzed by DLS and the histogram showed an average particle size (based on intensity distribution) at about 89 nm (Fig. 3). Moreover, TEM measurements showed well distribution of SiO_2_NPs without any agglomeration and the biosynthesized SiO_2_NPs are almost to be oval, round and cubic in shape (Fig. 4). The results of FTIR spectra of SiO_2_NPs confirmed the presence of various functional groups at about 3452.83, 2064.54 ,1634.67, 712.86 cm^−1^ corresponding to carbonyl residues, alcohol, nitrile, acid chloride, alkene band C—C stretch in–ring of CH_3_ , stretch of alkyl halides and peptide bonds of proteins responsible for the synthesis of the SiO_2_NPs (Fig. 5). The peaks were given along with the functional groups, which could be depicted to be 3452.83^-1^ associated with the OH bonds of the silanol groups and 1634.67^-1^nitro compounds (symmetrical stretch) and 712.86 cm^−1^ are which corresponding to C—Cl stretch of alkyl halides and to the symmetric and asymmetric Si—O—Si vibration, respectively.

### 3.3 Heavy metal concentration in water and fish samples

Cd, As, Cu, Fe, Hg, Pb, Mn and Zn concentrations in the water and tissue samples were presented in Table 1. Fe, Pb, Hg, Mn, As, Cu and Zn showed to the highest levels in the water samples respectively. Pb, Fe, Zn, Hg, As, Mn and Cu were the highest levels in the tissue samples respectively. The concentration of metals in water and tissues were higher in summer than other seasons. The recorded results indicated that Pb was the highest in the water (0.08848 mg/L) and muscles (5.535 mg/L).

**Table 1:** Mean value of heavy metals (ppm) in the Nile tilapia tissues and water samples from bahr el-baqar fish farm.

	**Water**				**P-Value**	**Sig**	**Fish Muscles**				**P-Value**	**Sig**
	Summer	Autumn	Winter	Spring			Summer	Autum	Winter	Spring		
**Cd(ppm)**	0.0504 ± 0.0060^a^	0.0076 ± 0.0006^c^	0.0002 ± 0.0001^c^	0.0265^b^ ± 0.0029^b^	0.000	**	0.2600 ± .01856^a^	0.0867 ± 0.0145^b^	0.0733 ± 0.0133^b^	0.1058 ± 0.0084^b^	0.000	**
**As(ppm)**	0.0573 ± 0.0278^a^	0.0025 ± 0.0002^b^	0.0009 ± 0.0007^b^	0.0083 ± 0.0002^b^	0.051	*	2.4300 ± 0.9114^a^	0.1050 ± 0.0150^b^	0.0879 ± 0.0011^c^	0.3948 ± 0.0195^b^	0.442	NS
**Cu(ppm)**	0.0173 ± 0.0023^a^	0.0080 ± 0.0011^b^	0.0010 ± 0.0001^c^	0.0159 ± 0.0014^a^	0.000	**	1.4967 ± 0.4822^a^	0.8367 ± 0.0984^b^	0.5400 ± 0.0900^b^	0.9767 ± 0.3638^a^	0.071	NS
**Fe(ppm)**	0.1294 ± 0.0593^a^	0.0072 ± 0.0011^b^	0.0037 ± 0.0004^b^	0.0138 ± 0.0081^b^	0.048	*	4.8800 ± 0.3329^a^	2.0200 ± 0.2909^bc^	0.94667 ± 0.0956^d^	3.7833 ± 0.8186^ab^	0.003	**
**Hg(ppm)**	0.0847 ± 0.0071^a^	0.0223 ± 0.0039^c^	0.0194 ± 0.0053^b^	0.0714 ± 0.0405^a^	0.317	Ns	4.2350 ± 0.7650^a^	0.8900 ± 0.0493^b^	0.5025 ± 0.0170^b^	0.8867 ± 0.0376^b^	0.000	**
**Pb(ppm)**	0.0885 ± 0.0004^a^	0.0388 ± 0.0032^c^	0.0221 ± 0.0014^c^	0.0664 ± 0.0013^b^	0.000	**	5.535 ± 0.1550^a^	0.90667 ± 0.0291^b^	0.5700 ± 0.0404^b^	3.8337 ± 0.9788^a^	0.004	**
**Mn(ppm)**	0.0629 ± 0.0009^a^	0.0050 ± 0.0040^b^	0.0057 ± 0.0026^b^	0.0095 ± 0.0007^b^	0.000	**	1.7500 ± 0.5800^a^	0.1667 ± 0.0598^b^	0.1067 ± 0.0233^b^	1.3650 ± 0.2974^a^	0.006	**
**Zn(ppm)**	0.0161 ± 0.0039^a^	0.0047 ± 0.0029^c^	0.0009 ± 0.0008^d^	0.0153 ± 0.0092^a^	0.176	NS	4.7167 ± 0.6754^a^	1.6075 ± 0.0659^b^	0.4780 ± 0.0220^b^	3.4067 ± 0.4788^a^	0.001	**

Values with different superscripts within rows are significantly different. Sample symbols (a:a) mean non significant differrence (a:b) mean significant difference.

** =P < 0.001

* =P < 0.05. NS = Not significant.

### 3.4 Adsorption capacity of Pb on SiO_2_NPs

**Fig. 6:**
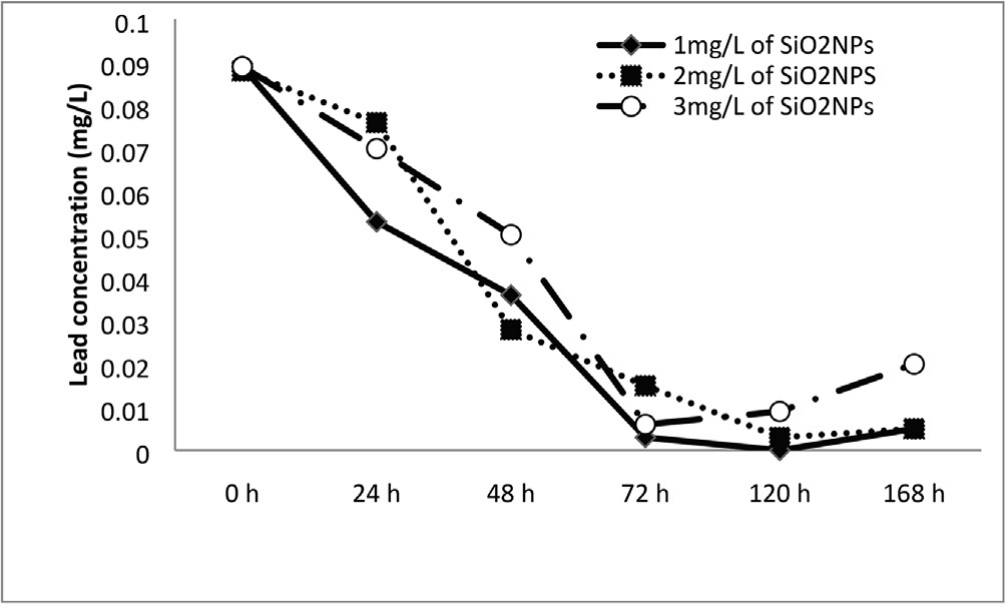
Adsorption capacity of SiO_2_NPs to lead through 168 h.

The results have showed that the concentration of Pb was decreased in water upon increasing the concentrations of SiO_2_NPs until 120 h; then a little increase in Pb concentration was noticed by further time until 168 h. The highest adsorption rate of Pb was observed at SiO_2_NPs concentration of 1 mg/L followed by 2 mg/L (Fig. 6). The adsorption capacity was enhanced by modification of SiO_2_NPs with surface functional groups and by increasing the contact time.

### 3.5 Clinical signs, postmortem findings of *O. niloticus*

**Fig. 7:**
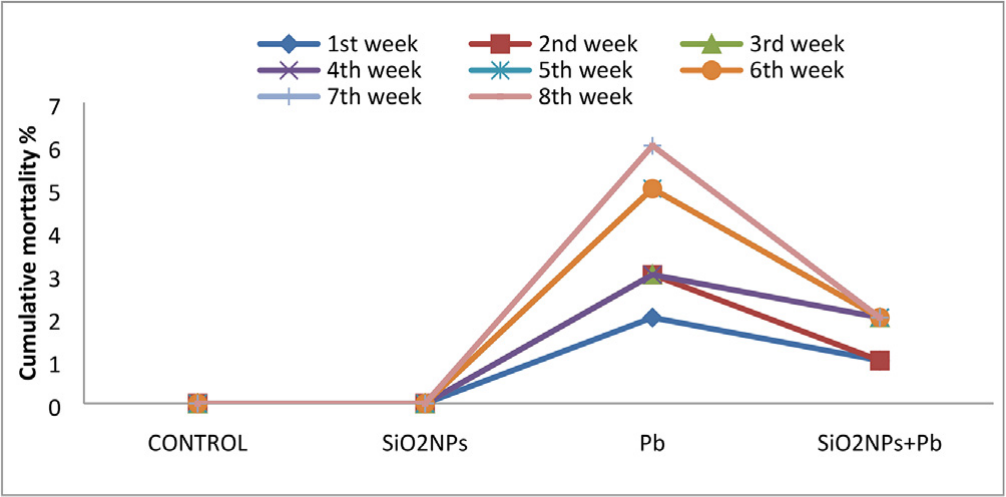
Effect of water lead toxicity remediation by SiO_2_NPs mediated by T. harzianum MF780864 on cumulative mortality of O. niloticus during experimental period (8 weeks).

The high mortality of *O. niloticus* was documented in the lead group followed by Pb + SiO_2_NPs (Fig. 7). During the experimental period, behavioral changes were only reported in the Pb-exposed group. At the beginning of the experiment, the fish samples in the Pb-exposed group were hyperactive with restlessness. Then include hematological, neural disorders and tetanic spasms together with some morphological changes such as darkening in caudal fin and covering of the gills by a mucus layer and some cases ended by fish mortality.

### 3.6 Growth performance of *O. niloticus*

The results of growth performance (FBW, WG, FCR and SGR) of *O. niloticus* are presented in Table 2. Result have showed that SiO_2_NPs and SiO_2_NPs + Pb increased final body weight (FBW), weight gain (WG) and specific growth rate (SGR) but decreased feed conversion ratio (FCR) compared to lead (pb) treatment.

**Table 2:** Effect of water lead toxicity remediation by SiO_2_NPs mediated by T. harzianum MF780864 on O. niloticus growth performance after 8 weeks.

**Treatment**	**CONT**	**SiO_2_NPs**	**Pb**	**SiO_2_NPs + Pb**	**P-Value**	**sig**
**IBW (g)**	29.70 ± 0.02	29.77 ± 0.14	29.82 ± 0.06	29.83 ± 0.02	0.595	NS
**FBW (g)**	53.24 ± 0.34^a^	52.18 ± 0.50^a^	47.72 ± 0.34^b^	51.84 ± 0.69^a^	0.000	**
**WG (g)**	23.54 ± 0.36^a^	22.42 ± 0.37^a^	17.90 ± 0.29^b^	22.01 ± 0.72^a^	0.000	**
**FCR**	2.09 ± 0.001^b^	2.16 ± 0.08^b^	2.52 ± 0.01^a^	2.13 ± 0.02^b^	0.000	**
**SGR**	0.97 ± 0.01^a^	0.94 ± 0.01a^b^	0.78 ± 0.01^c^	0.92 ± 0.02^b^	0.000	**

Values with different superscripts within rows are significantly different (P < 0.05). NS:Not significant. IBW:initial body weight. FBW:final body weight. WG:weight gain. FCR: feed conversion ratio. SGR: specific growth rate. Sample symbols (a:a) mean non significant differrence (a:b) mean significant difference.

### 3.7 Hematological parameters and biochemical parameters of *O. niloticus*

The results of biochemical blood parameters are given in Table 3. Exposure to Pb resulted in significant changes in all the hematological indices, including a significant reduction in RBC count, Hb, Hct, and MCHC. These values were significantly modulated in SiO_2_NPs + Pb co-treated group, where such indices depicted a significant increase in RBC count, Hb, Hct, and MCHC. The level of improvement achieved by the co-exposure did not match with the values of estimated indices obtained in the control group. Regarding the number of WBCs, lymphocytes, and neutrophils, they significantly decreased in the Pb-exposed group followed by the Pb + SiO_2_NPs group, compared with other groups not exposed to Pb.

**Table 3:** Effect of water lead toxicity remediation by SiO_2_NPs mediated by T. harzianum MF780864 on O. niloticus hematological parameters after 8 weeks.

**Treatment**	**CONTROL**	**SiO_2_NPs**	**Pb**	**SiO_2_NPs + Pb**	**P-Value**	**Sig**
**Red Blood Cells**						
**RBCs(10^6^/ml)**	2.92 ± 0.12^a^	2.90 ± 0.06^a^	1.89 ± 0.19^c^	2.44 ± 0.07^b^	0.001	*
**Hb(g/dl)**	9.27 ± 0.29^a^	8.97 ± 0.33^a^	6.57 ± 0.54^b^	9.70 ± 0.058^a^	0.001	**
**Hct(%)**	32.67 ± 1.45^a^	32.33 ± 0.33^a^	22.33 ± 3.18^b^	29 ± 0.2.31^ab^	0.028	*
**MCV(fl)**	111.96 ± 0.44	111.45 ± 2.41	108.99 ± 0.58	109.58 ± 1.29	0.430	NS
**MCHC(g/dl)**	28.40 ± 0.47^a^	27.74 ± 1.13^a^	23.25 ± 1.50^b^	26.04 ± 0.59^ab^	0.028	*

**White Blood Cells**						
**WBCs**	7.16 ± 0.18^a^	6.01 ± 0.16^b^	5.14 ± 0.21^c^	5.15 ± 0.17^c^	0.000	**
**Lymphocyte**	3.84 ± 0.14^a^	3.31 ± 0.21^b^	2.49 ± 0.14^c^	2.81 ± 0.05^c^	0.001	**
**Heterophil**	1.82 ± 0.09	1.51 ± 0.10	1.50 ± 0.08	1.53 ± 0.11	0.113	NS
**Eosinophil**	0.38 ± 0.01	0.36 ± 0.01	0.35 ± 0.02	0.34 ± 0.02	0.650	NS
**Monocyte**	1.12 ± 0.09^a^	0.82 ± 0.06^b^	0.79 ± 0.01^b^	0.71 ± 0.02^b^	0.003	*

Values with different superscripts within rows are significantly different.

* =P < 0.05. NS = Not significant. RBCs = Red blood cells. Hb = Hemoglobin. Hct = The hematocrit. MCV = Mean corpuscular volume. MCHC = Mean corpuscular hemoglobin concentration. WBCs = White blood cells.

** =P < 0.001

**Fig. 8:**
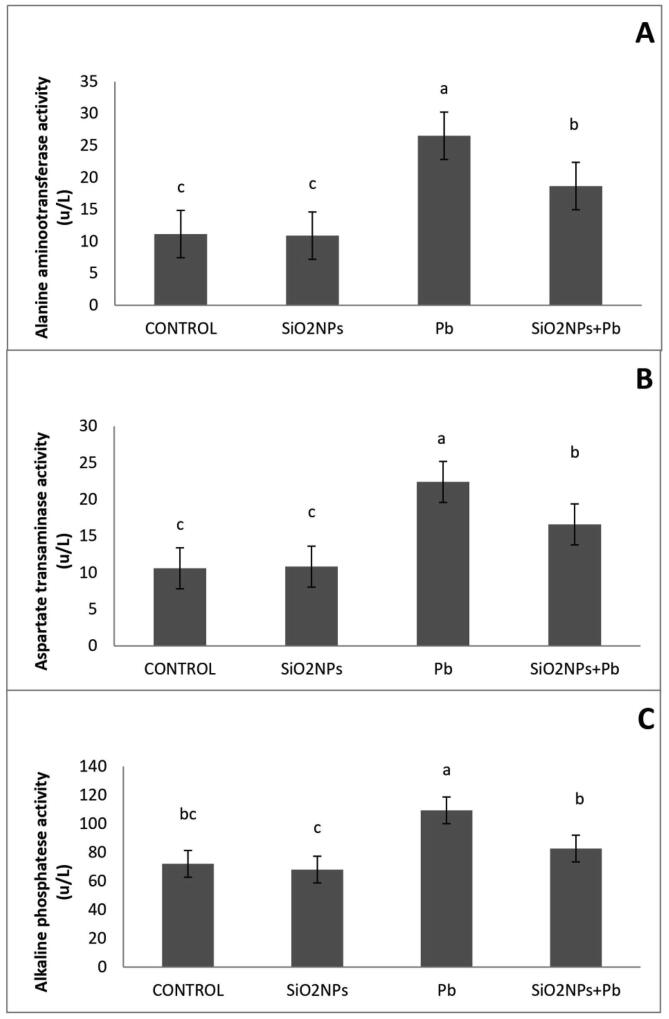
Effect of water lead toxicity remediation by SiO_2_NPs mediated by T. harzianum MF780864 on O. niloticus liver function after 8 weeks. (A). Bars indicate alanine amino transferase (ALT), (B). Bars indicate Aspartate Aminotransferase (AST). (C). Bars indicate Alkaline phosphatase (ALP). Groups with different superscripts (a, b and c) are significantly different (P < 0.05, using a one-way ANOVA)**.**

**Fig. 9:**
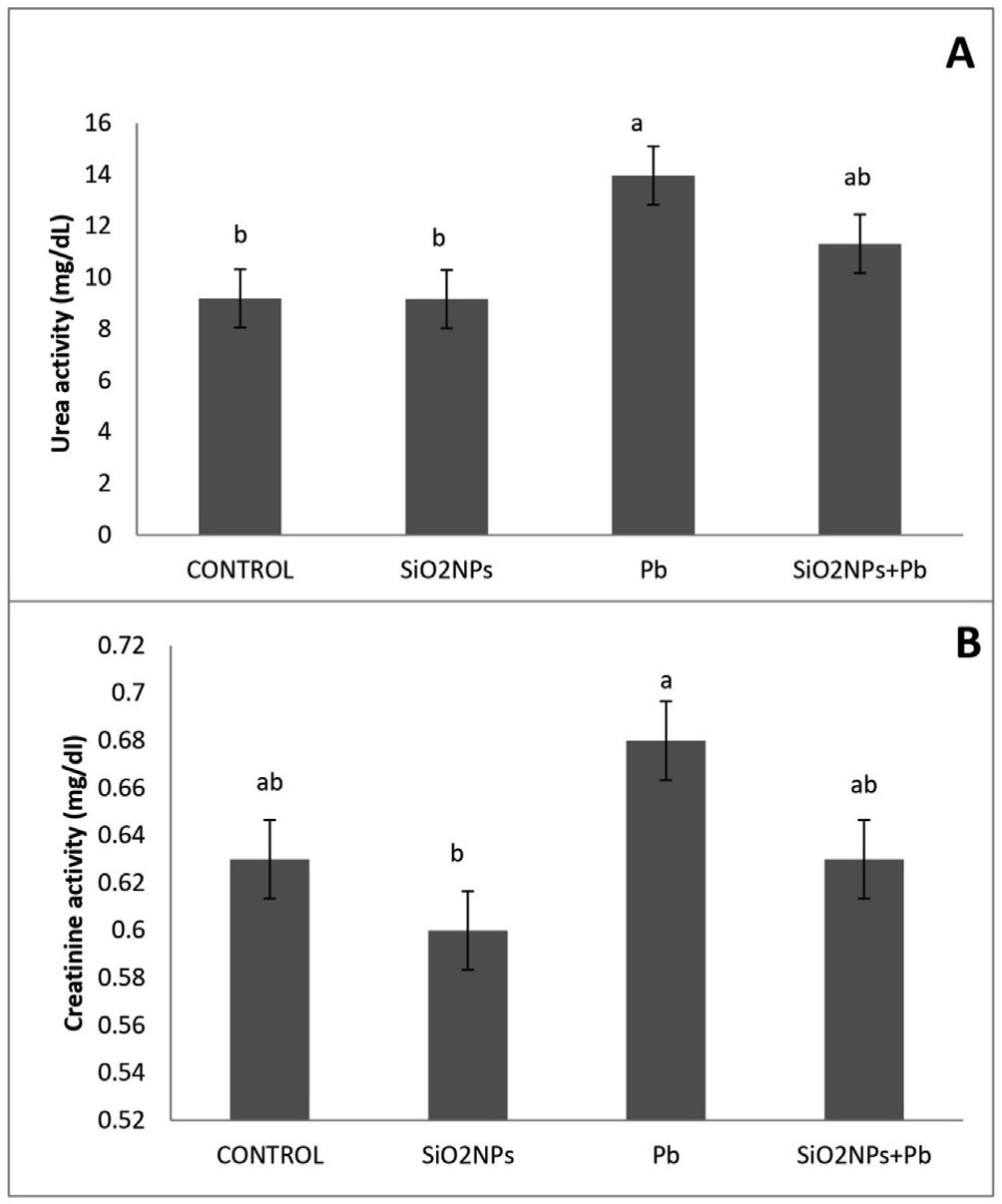
Effect of water lead toxicity remediation by SiO_2_NPs mediated by *T. harzianum* MF780864 on *O.s niloticus* kidney function after 8 weeks. (A). Bars indicate urea. (B). Bars indicate creatinine. Groups with different superscripts (a, b and c) are significantly different (P < 0.05, using a one-way ANOVA)**.**

Effect of water lead toxicity remediation by SiO_2_NPs onO. niloticus liver functions after 8 weeks were studied (Fig. 8). It was shown that the fish lead exposure increased liver functions as AST, ALT and ALP activity were of the highest values as compared to control group. In addition, such indices revealed a significant decrease in the SiO_2_NPs + Pbco-treated group; the modulated indices did not reach values recorded in the control group. Fish treated with Pb exhibited the highest values of urea and creatinine activity as compared to other treatments (Fig. 9).

### 3.8 Oxidant/antioxidant capacity of *O. niloticus*

**Fig. 10:**
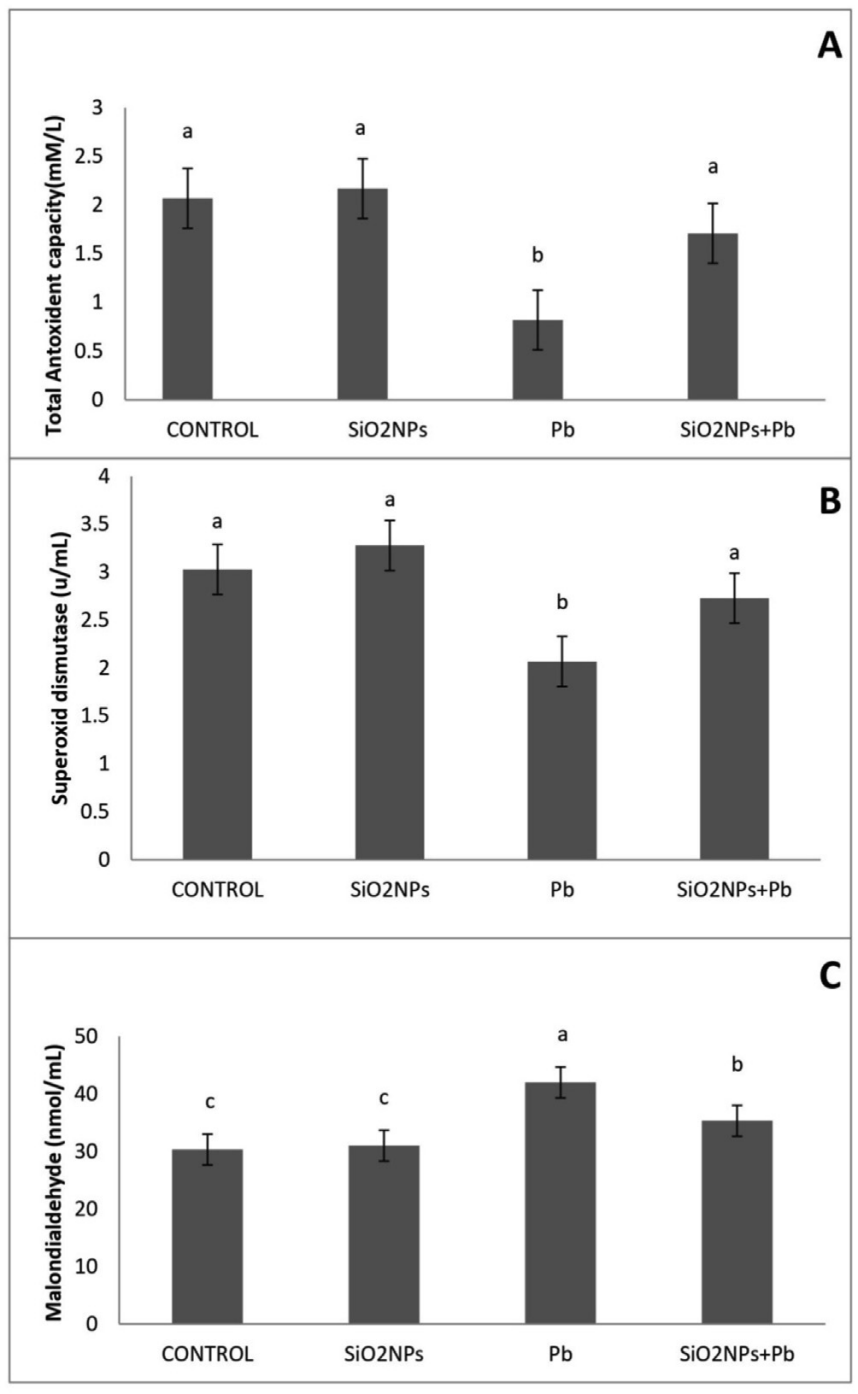
Effect of water lead toxicity remediation by SiO_2_NPs mediated by *T.harzianum* MF780864 on *O. niloticus* antioxidant status after 8 weeks. A). Bars indicate total antioxidant capacity (TAC), B). Bars indicate Superoxide dismutase (SOD). C). Bars indicate Malondialdehyde (MDA). Groups with different superscripts (a, b and c) are significantly different (P < 0.05, using a one-way ANOVA)**.**

The effect of SiO_2_NPs on antioxidant responses of *O. niloticus* are given in Fig. 10. A significant increase was recorded in TAC and SOD levels in the SiO_2_NPs-exposed groups compared to lead treatment only. On the other hand, a significant increase was recorded in MDA levels in the Pb-exposed group, whereas the control and SiO_2_NPs groups showed noticeably decreased levels.

### 3.9 Non-specific immune parameters of *O. niloticus*

**Fig. 11:**
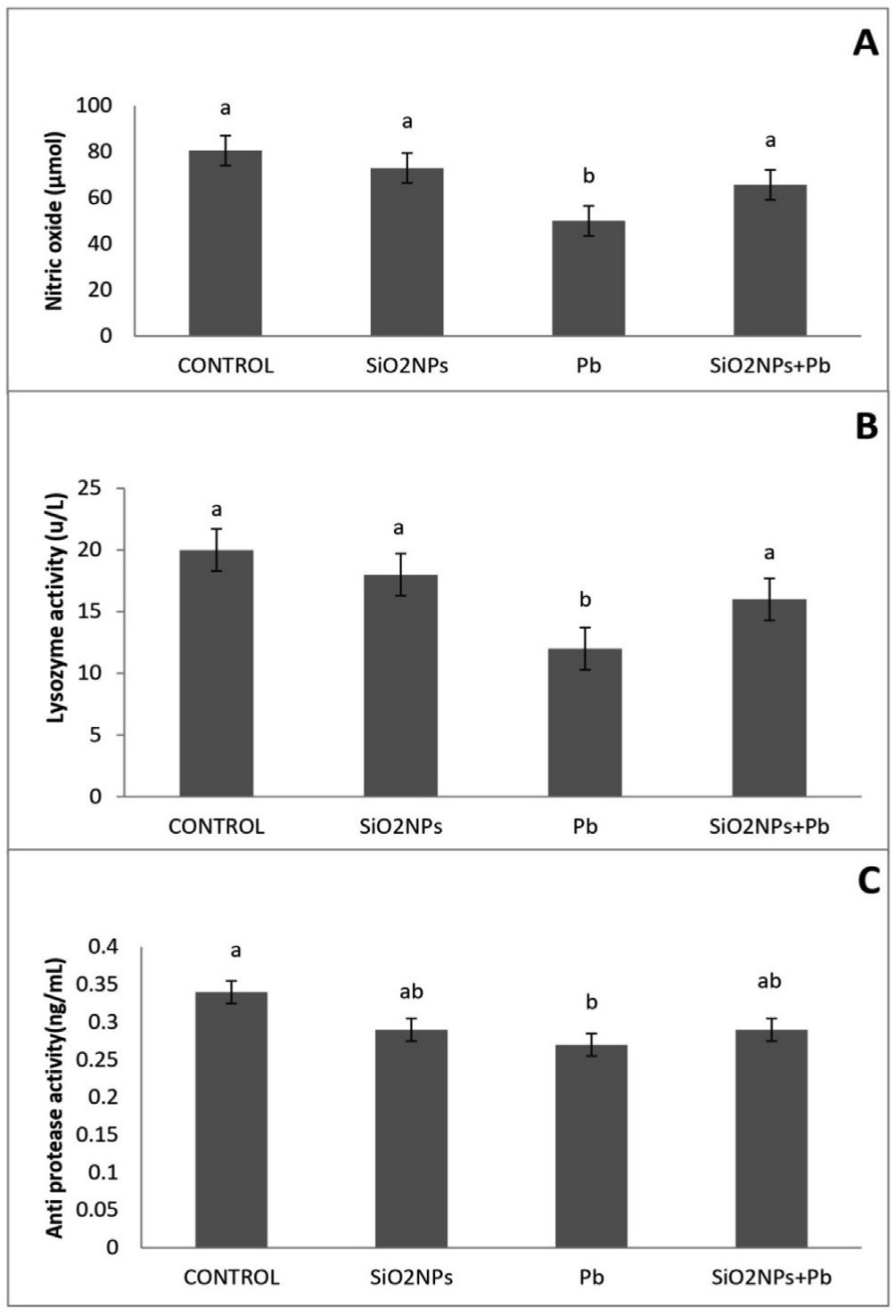
Effect of water lead toxicity remediation by SiO_2_NPs mediated by *T. harzianum* MF780864 on *O. niloticus* non-specific immune parameters after 8 weeks. A). Bars indicate nitric oxide activity (NO). B) Bars indicate lysozyme activity (LYZ). C). Bars indicate antiprotease activity (APA). Groups with different superscripts (a and b) are significantly different (P < 0.05, using a one-way ANOVA)**.**

The effect of Pb exposure and/or SiO_2_NPs supplementation on immune parameters was shown in Fig. 11. Exposure to Pb resulted in significant changes in all the immune parameters, including a significant reduction in NO, LYZ and APA compared to all treatment. SiO_2_NPs improved the recorded decrease in LYZ, NO and APA activity of the Pb-exposed group.

### 3.10 Relative expression level of immune related Il-1β

**Fig. 12:**
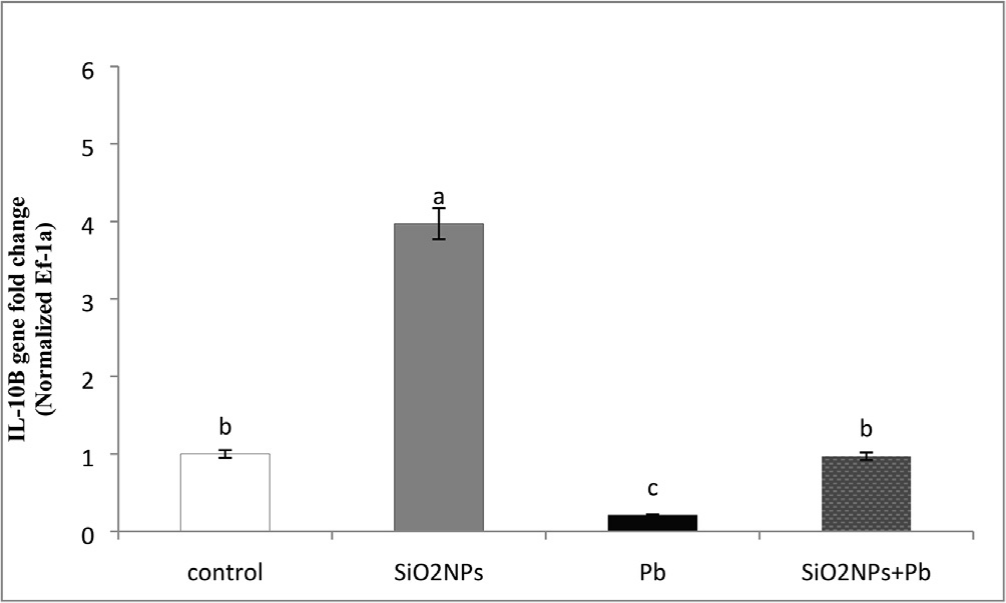
Effect of water lead toxicity remediation by SiO_2_NPs mediated by *T. harzianum* MF780864 on quantitative RT (real time)-PCR gene expression of IL-1β of *O. niloticus* after 8 weeks. Groups with different superscripts significantly different (P < 0.05, using a one-way ANOVA)**.**

Water lead toxicity remediation by SiO_2_NPsthrough immune related gene expression of cytokines IL-1*β* of O*. niloticus* was presented in Fig. 12. The immune related *Il-1β* expression was significantly higher in the fish groups that treated with SiO_2_NPs supplementation than Pb exposed group by about 4 fold. In addition, the presence of SiO_2_NPs with Pb exposed group enhanced the immune IL1B expression by about 2 fold compared with single Pb exposed group.

### 3.11 Lead residues in muscular of *O. niloticus*

**Fig. 13:**
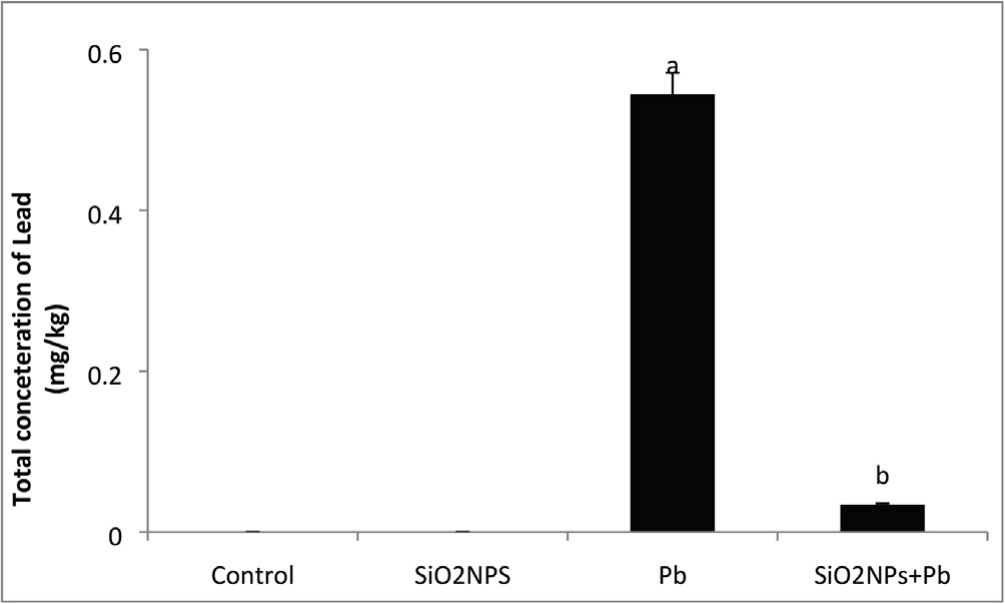
Effect of SiO_2_NPs size on level of lead (Pb) residues in musculature of *O. niloticus* exposed to lead toxicity (0.088 mg/L) for 60 days.

The Pb residue concentration in muscles of O. niloticus is shown in Fig. 13, which clarifies the significant decrease of Pb concentration in muscles of fish treated by SiO_2_NPs compared with Pb exposed group without any treatment.

## 4 Discussion

Heavy metals such as Pb causes water toxicity and are one of certain risks for human health as they are toxic and carcinogenic (Rahman et al., 2012, Masindi and Muedi, 2018, Lee et al., 2019). Hence, this study was an endeavor to reduce Pb concentration of polluting water by its absorption by SiO_2_NPs synthesized by T. harzinum; this is to concur with recent prospective of using nanotechnology in improving the safety of aquaculture ecosystems (Ahmed et al., 2017, Kotsyuda et al., 2017, Chris et al., 2018, Enan et al., 2020). This method is one of the highly efficient HMs remediation protocols that are easily applicable and of low cost, especially using eco-friendly plant wastes such as RHs (Chiban et al., 2012).

It was showed herein that a fungal isolate that identified as T. harzinum; can biosynthesize SiO_2_NPs using RHs as approved by UV–vis spectra at about 336 nm and this is coupled with previous published results (El-Gazzar et al., 2020). A single peak of about 89 nm was showed by DLS technique; indicating on the purity and quality of the biosynthesized SiO_2_NPs and such result supports the findings of Nakkala et al. (2017). The biosynthesized SiO_2_NPs herein were well dispersed in the solution without agglomeration, because fungal metabolites include various biomolecules which could be responsible for synthesis and stabilization of these SiO_2_NPs and could also be used as capping agents for preventing their agglomeration; these biomolecules may be peptides, phenolic compounds, proteins or carbohydrates (El-Gazzar and Ismail, 2020). Various functional groups were elucidated herein by FTIR analysis and such result reflected the presence of various biomolecules in fungal metabolites which could encapsulate SiO_2_NPs and increase their stability whereas the associated proteins might be used in mineralization of the precursor RHs (El-Gazzar and Ismail, 2020).

It was showed herein that the HMs such as Pb, Hg, and Cd were of high levels in the collected water and fish samples; indicating on the pollution of these samples. In addition, these HMs were higher in summer than in other seasons because high temperature in summer (≥35 ^o^C in Egypt) stimulate growth of aquatic microflora which decompose organic wastes giving HMs (Rahman et al., 2012). In a latter study (Hamed et al., 2011, Sivakami et al., 2013), it was approved also that the samples provided from such area under study (Sahl AL Hussainia, Bahr El-Baqar, Egypt) were showed to be polluted by HMs. Pb concentration appeared in this study (0.088 mg/L) is above the permissible limits (0.001 ppm) set by the Egyptian organization standards (EOS, 2010); this showed that there is a mandatory need to search for decreasing such high levels of HMs by nanotechnology strategies.

The optimum concentration of SiO_2_NPs adopted in this study for adsorption of Pb was of about 1 mg/L and such result concur with latter published results (Afroze and Sen, 2018, Farooghi et al., 2018). In the present study, Pb concentration was decreased with the increased SiO_2_NPs concentration until 120 h, and then it was increased again at 168 h; this is could be due to the adsorptive power of the wide surface area of SiO_2_NPs which reached its saturation by Pb after (120 h). This result coincided with the findings of a previous authors (Farooghi et al., 2018) who clarified that the wide surface area of SiO_2_NPs enables high adsorption of a large amount of Pb at the first 120 h. with time, the chelation of HMs is mainly affected by the pH range, wherein the carboxyl acidic groups of the chelating agents are dissociated in a slightly alkaline medium by increasing the contact time. So, the adsorption capacity can be enhanced by modification of SiO_2_NPs with surface functional groups by increasing the contact time and this is coincided with latter published results (Nguyen et al., 2019).

FTIR spectra revealed an adsorption of different functional groups of phenolic compounds, protein and enzymes that increase the SiO_2_NPs chelation to the HMs, similarly to that reported in a previous work (Manyangadzea et al., 2020). Moreover, the richness of SiO_2_NPs by the oxygenated surface groups confirmed by FTIR increases the SiO_2_NPs chelation to the HMs including Pb (Ben-Ali et al., 2017, El-Gazzar and Ismail, 2020). In addition, the presented data confirmed that the small size with large surface area and stability of SiO_2_NPs by FTIR play an important role in Pb adsorption, hence increasing the chelation of SiO_2_NPS to Pb similarly to that reported by El-Gazzar and Enan. (2020) who reported that nanotechnology offers the ability to control matter at the nanoscale to have unique merits through their small diameter and large surface area.

The results in the present study showed that SiO_2_NPs and SiO_2_NPs + Pb improved FBW, WG, SGR and FCR compared to lead treatment, similarly with that reported previously (Srinivasan et al., 2016); Wherein the exposure to Pb evoked a significant decline in most of the hematological variables (RBC count, Hb, Hct, and MCHC) compared to SiO_2_NPs + Pb. These results indicated that the use of SiO_2_NPs in *O. niloticus* was safe and is capable of improving the body functions (Onuegbu et al., 2018).

Liver functions profile such as AST, ALT and ALP within serum samples of blood withdrawn from *O.niloticus* fed in water provided with Pb only were highly above normal level; similarly to that reported previously in this respect(Bitiren et al., 2004, Hodson et al., 1980) and more than that obtained from *O.niloticus* cultivated in H_2_O supplemented with Pb + SiO_2_NPs; indicating on the improvement of liver functions of fish samples containing SiO_2_NPs; similarly to that reported by Ashouri et al, (2015) who confirmed that the supplementation of SiO_2_NPs at the rate of 0.5 mg/kg exerted positive effects on the liver status of the crap**.** On contrary, the supplementation with SiO_2_NPs at the rate of 2 mg/kg exerted negative effects on the liver status of the crap (Ashouri et al., 2015). In the same framework, Pb treatment of *O. niloticus* exhibited elevation of kidney functions (Urea, creatinine) above their normal levels; indicating on kidneys dysfunction caused by Pb toxicity and the presence of SiO_2_NPs plus Pb protect *O. niloticus* from this Pb toxicity as urea and creatinine levels were normal; similarly to the results published previously in this respect(Hodson et al., 1980). This could be due to the absorptive capacity of Pb by SiO_2_NPs.

In the present study, the activities of liver TAC and MDA were significantly increased at using 1 mg/L SiO_2_NPs, similarly to Ashouri et al.(2015) who reported that supplementation of fish samples with 0.5 mg SiO_2_NPs /kg seems to be more effective than lower levels of SiO_2_NPs in strengthening the antioxidant system against oxidative stress. Moreover, the effect of SiO_2_NPs supplementation improves immune parameters that concur with the previous reports (Atencio et al., 2009, Awad et al., 2020). The expression level of *Il-1β* in the current study may be attributed to the activation of SiO_2_NPs, which is extremely important with regard to enhancing the entire immunity system including both the innate and adaptive immune response similarly to that reported previously (Enan et al., 2014, Ahmed et al., 2019, Awad et al., 2020, El-Gazzar et al., 2020). Muscle tissues are commonly used in studies that monitor the bioavailability of Pb pollution to the food web (Ahmed et al., 2019). From the data obtained, the Pb residue level in the muscles was decreased in SiO_2_NPs + Pb treated groups and this result similar with findings of other authors Abdelazim et al, (2018) who reported that the applying vitamin C had significantly decreased the levels of oxidative stress in *O. niloticus* muscles.

Further work will be necessary to study the biosorption of Pb by other nanoparticles in vitro and in vivo and to carry out research work on the effect of Pb on cell organelles

## 5 Conclusions

One fungal isolate that identified as *T. harzianum* showed a promising capability to biosynthesis SiO_2_NPs using RHs as a cheap substrate. The obtained SiO_2_NPs were characterized herein using the possible instrumental analysis such as UV, DLS, FTIR and TEM. The obtained SiO_2_NPs decreased Pb concentration from either water or *O.niloticus* muscles as they adsorbed this toxic Pb metal at concentration of about 1 mg/L, hence improve liver, kidney functions and immune parameters of *O. niloticus*. Moreover, SiO_2_NPs increased immune related IL-B gene of *O. niloticus* and improved this gene upon the existence of Pb in water.

## Funding

Non Funding.

## CRediT authorship contribution statement

**Nashwa El-Gazzar:** Conceptualization, Methodology, Software, Validation, Formal analysis, Investigation, Resources, Data curation, Writing - original draft, Writing - review & editing, Supervision, Project administration. **Taghreed N. Almanaa:** Writing - original draft, Funding acquisition, Validation, Formal analysis. **Rasha M. Reda:** Conceptualization, Software, Validation, Formal analysis, Investigation, Resources, Data curation, Project administration. **M.N. El Gaafary:** Conceptualization, Supervision, Project administration. **A.A. Rashwan:** Conceptualization, Writing - review & editing, Supervision, Project administration. **Fatma Mahsoub:** Software, Formal analysis.

## Declaration of Competing Interest

The authors declare that they have no known competing financial interests or personal relationships that could have appeared to influence the work reported in this paper.
