# Corrigendum to “Assessment the using of silica nanoparticles (SiO2NPs) biosynthesized from rice husks by *Trichoderma harzianum* MZ066609 as water lead adsorbent for immune status of Nile tilapia (*Oreochromis niloticus*)” [Saudi J. Biol. Sci. 28(9) (2021) 5119–5130]

**DOI:** 10.1016/j.sjbs.2022.02.003

**Published:** 2022-02-15

**Authors:** Nashwa El-Gazzar, Taghreed N. Almanaa, Rasha M. Reda, M.N. El Gaafary, A.A. Rashwan, Fatma Mahsoub

**Affiliations:** a Department of Botany and Microbiology, Faculty of Science, Zagazig University, 44519 Zagazig, Egypt; b Department of Botany and Microbiology, College of Science, King Saud University, 11495 Riyadh, Saudi Arabia; c Faculty of Veterinary Medicine, Department of Fish Diseases and Management, Zagazig University, 44511 Zagazig, Egypt; d Faculty of Technology and Development, Department of Animal & Poultry Production, Zagazig University, 44519 Zagazig, Egypt

Please correct the accession number of *Trichoderma harzianum* MF780864 into *Trichoderma harzianum* MZ 066609.

  This corrigendum presents in text at the following parts:

1. **In Title of article:**

From:

Assessment the using of silica nanoparticles (SiO2NPs) biosynthesized from rice husks by *Trichoderma harzianum* MF780864 as water lead adsorbent for immune status of Nile tilapia (*Oreochromis niloticus*)

Into:

Assessment the using of silica nanoparticles (SiO2NPs) biosynthesized from rice husks by *Trichoderma harzianum* MZ066609 as water lead adsorbent for immune status of Nile tilapia (*Oreochromis niloticus*)  

2. **Abstract, Line 2:**

From:

An isolate of *Trichoderma harzianum* MF780864 (T. harzianum) was isolated and identified based on the Internal Transcribed Spacers (ITS) sequences;

Into:

An isolate of *Trichoderma harzianum* MZ066609 (*T. harzianum*) was isolated and identified based on the Internal Transcribed Spacers (ITS) sequences;  

3. **In the title of Figure no (1):**

From:

Fig. 1. Phylogenetic tree based on ITS sequences of 18S-28S rRNA of the fungal strain (sample _1 *Trichoderma harzianum*. MF780864).

Into:

Fig. 1. Phylogenetic tree based on ITS sequences of 18S-28S rRNA of the fungal strain (sample _1 *Trichoderma harzianum*. MZ066609).  

4. **Result section, subsection (3.1) Line (24) Page (5122)**

From:

Hence, the isolate of *Trichoderma harzianum* was designed as FM-*Trichoderma harzianum* MF780864.

Into:

Hence, the isolate of *Trichoderma harzianum* was designed as FM-*Trichoderma harzianum* MZ066609.

  The author would like to apologize for any inconvenience caused.

