# Supplementary material for: Assessment the using of silica nanoparticles (SiO2NPs) biosynthesized from rice husks by Trichoderma harzianum MF780864 as water lead adsorbent for immune status of Nile tilapia (Oreochromis niloticus)
Source: Saudi J Biol Sci. 2021 May 21;28(9):5119–30. doi: 10.1016/j.sjbs.2021.05.027 (PMC8381041; doi:10.1016/j.sjbs.2021.05.027)
Supplement: Supplementary data 1 [file mmc1.docx]

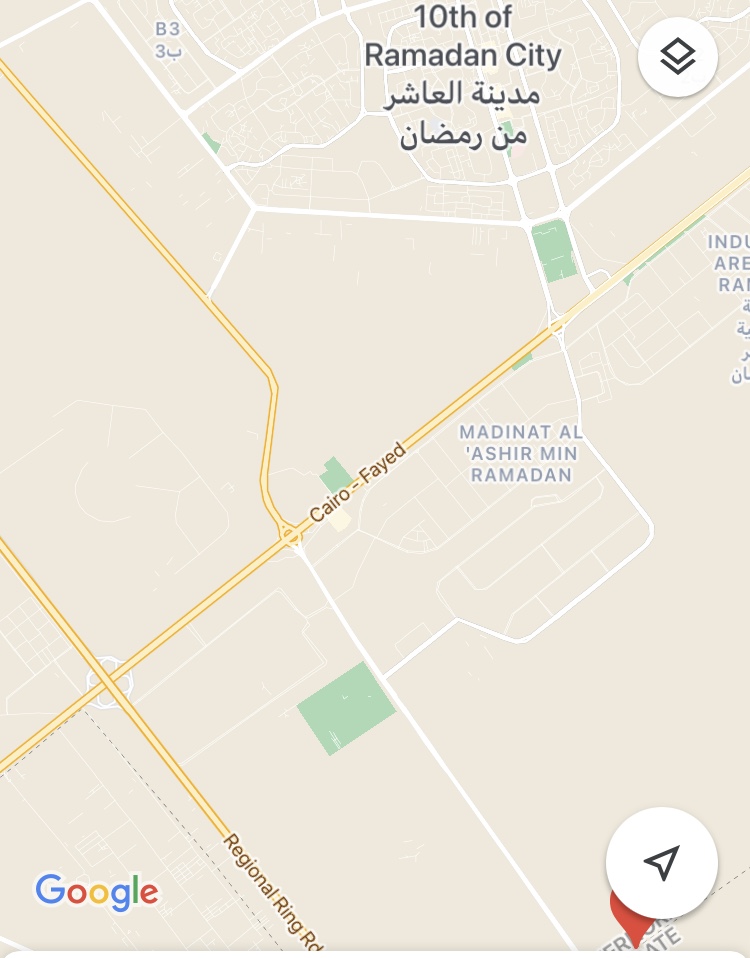


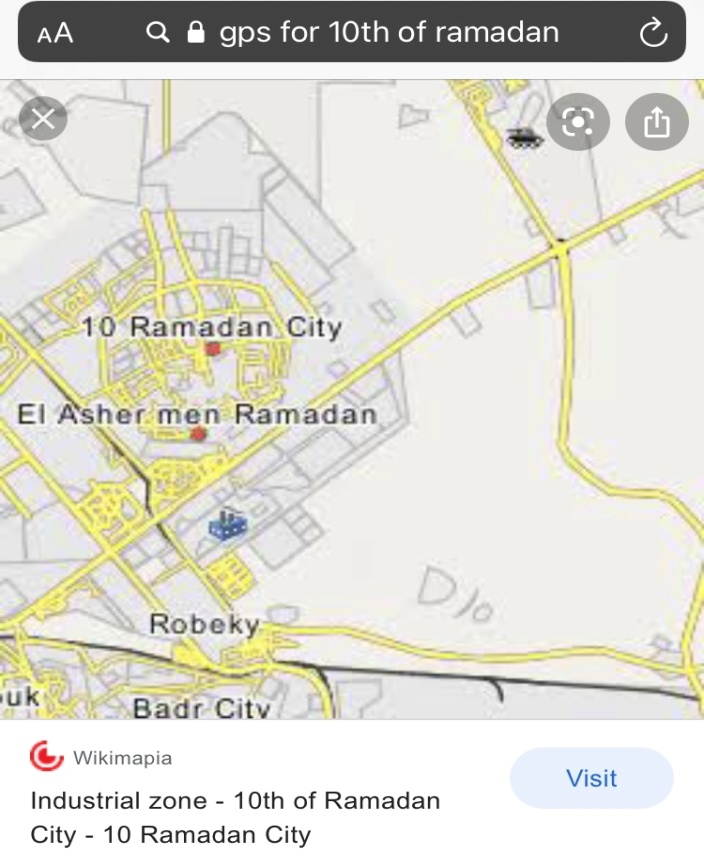

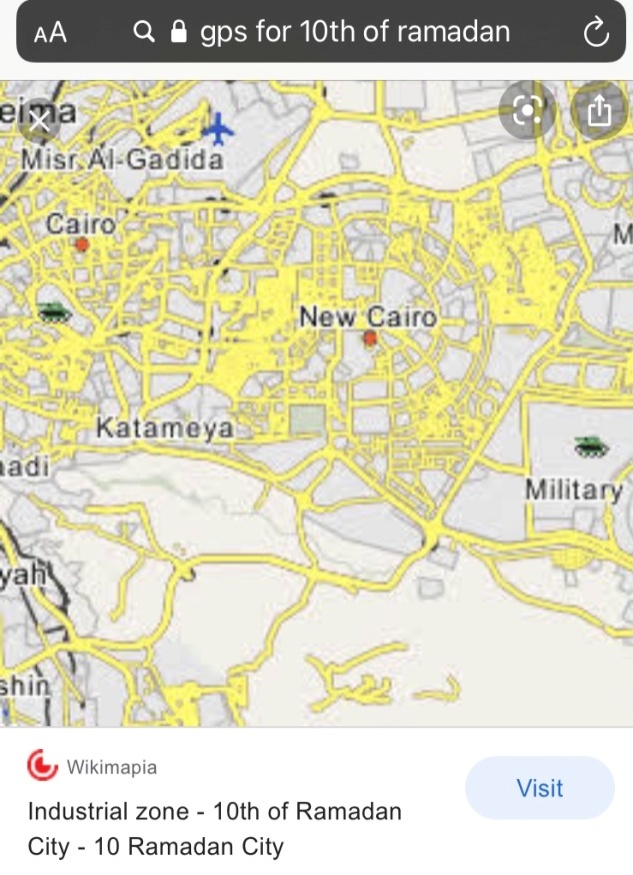


**Supplementary Fig.1.** GBS location of the isolation area for 10th of Ramadan City is given herein. It is located 20 km in the Eastern North side of Cairo, Egypt.


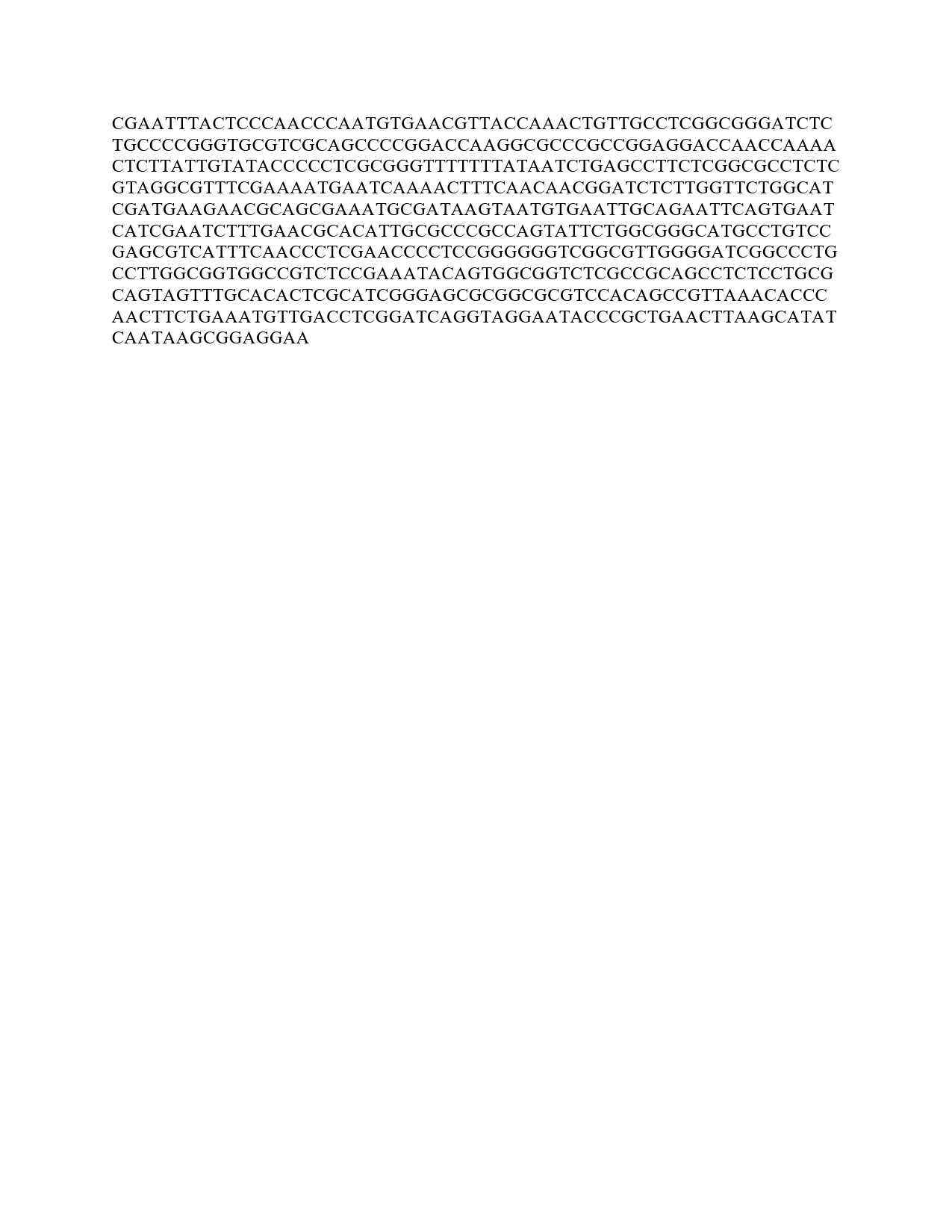


**Supplementary Fig.2.**Sequence of 18S-28Sr RNA region of *Trichoderma harzianum* genome (593bp)
